# Vowel acoustics of Nungon child-directed speech, adult dyadic conversation, and foreigner-directed monologues

**DOI:** 10.3389/fpsyg.2022.805447

**Published:** 2022-09-13

**Authors:** Hannah S. Sarvasy, Weicong Li, Jaydene Elvin, Paola Escudero

**Affiliations:** ^1^MARCS Institute for Brain, Behaviour, and Development, Western Sydney University, Penrith, NSW, Australia; ^2^Australian Research Council (ARC) Centre of Excellence for the Dynamics of Language, Canberra, ACT, Australia; ^3^Department of Linguistics, California State University, Fresno, CA, United States

**Keywords:** Nungon, acoustics, vowel, child-directed speech, foreigner-directed speech, hypo-articulation, hyper-articulation, prosody

## Abstract

In many communities around the world, speech to infants (IDS) and small children (CDS) has increased mean pitch, increased pitch range, increased vowel duration, and vowel hyper-articulation when compared to speech directed to adults (ADS). Some of these IDS and CDS features are also attested in foreigner-directed speech (FDS), which has been studied for a smaller range of languages, generally major national languages, spoken by millions of people. We examined vowel acoustics in CDS, conversational ADS, and monologues directed to a foreigner (possible FDS, labeled MONO here) in the Towet dialect of the Papuan language Nungon, spoken by 300 people in a remote region in northeastern Papua New Guinea. Previous work established that Nungon CDS entails optional use of consonant alteration, special nursery vocabulary, and special morphosyntax. This study shows that Nungon CDS to children aged 2;2–3;10 lacks vowel hyper-articulation, but still displays other common prosodic traits of CDS styles around the world: increased mean pitch and pitch range. A developmental effect was also attested, in that speech to 2-year-olds contained vowels that were significantly longer than those in speech to 3-year-olds, which in turn had vowels of similar duration to those in Nungon ADS. We also found that Nungon FDS vowel triangles, measured from monologues primarily directed to a non-native speaker, were significantly larger than those of either CDS or conversational ADS, indicating vowel hyper-articulation. The Nungon pattern may align with the patterns of vowels in Norwegian IDS, CDS, and FDS, where hyper-articulation is found in FDS, but not CDS or IDS. The languages of the New Guinea area constitute 20% of the world's languages, but neither an acoustic comparison of vowels in CDS and ADS, nor an acoustic study of FDS, has previously been completed for any language of New Guinea. The function of an FDS style in a small, closed community like those of much of New Guinea may differ from that in larger societies, since there are very few non-native speakers of Nungon. Thus, this study uses monologues recorded with a foreign researcher as interlocutor to study Nungon FDS.

## Introduction

In many communities around the world, speech directed at infants (IDS) and young children (CDS) involves special acoustic and prosodic features, compared with adult-directed speech (ADS). Among the special acoustic and prosodic features frequently attested in IDS/CDS styles are: increased mean pitch and increased pitch range (Fernald et al., 1989), longer vowel durations (Swanson et al., 1992 found this especially for English content words, not function words), and vowel hyper-articulation, usually understood to involve an expanded vowel space (most often calculated using the first and second formant frequencies of the vowels /i/, /a/, and /u/; Kuhl et al., 1997; Burnham et al., 2002; Uther et al., 2007; Lam and Kitamura, 2008). These acoustic and prosodic modifications may change with children's age and development, as seen, for instance, in differences in caregiver vowel spaces in Cantonese IDS to children of different ages (Stern et al., 1983; Kitamura et al., 2002; Kitamura and Burnham, 2003; Englund and Behne, 2006; Rattanasone et al., 2013). The magnitude of increase in CDS mean pitch and pitch range, relative to ADS, may decrease after the first year or two of life (Garnica, 1977; Stern et al., 1983; Warren-Leubecker and Bohannon, 1984), but Warren-Leubecker and Bohannon (1984) found that American English-speaking mothers (but not fathers) still used elevated pitch in CDS to 5-year-old children.

Perhaps the most controversial proposed feature of IDS/CDS, with the greatest number of counter-examples in the literature, is vowel hyper-articulation (Cristia and Seidl, 2014). The IDS/CDS vowel space has been found to be larger than in adult-directed speech (ADS) for: American, Australian, and British English (Kuhl et al., 1997; Burnham et al., 2002; Uther et al., 2007; but see Green et al., 2010), Russian (Kuhl et al., 1997), Mandarin (Liu et al., 2003), Spanish (García-Sierra et al., 2021), Swedish (Kuhl et al., 1997; but see Van de Weijer, 2001), and Japanese (Andruski et al., 1999; but see Martin et al., 2015; Miyazawa et al., 2017). However, for other languages, not only has no expansion of the vowel space in IDS/CDS relative to ADS been demonstrated, but rather a reduction of the vowel space has been shown. For instance, Rattanasone et al. (2013) found that the vowel triangle (formed from the three “corner” vowels, /i/, /a/, and /u/) for Cantonese speech addressed to 3-month-old infants was significantly smaller than that for ADS, suggesting hypo-articulation of IDS vowels at that stage. A marked reduction of the vowel space in IDS/CDS relative to ADS is also attested for Dutch (Benders, 2013), German (Audibert and Falk, 2018), and Norwegian (Englund and Behne, 2006).

Postulated functions of the special acoustic and prosodic features of IDS and CDS can be divided into three main categories (Grieser and Kuhl, 1988; Cooper et al., 1997; Singh et al., 2002; Uther et al., 2007): (a) obtaining infants'/children's attention (especially through expanded pitch range; Fernald and Simon, 1984); (b) expressing positive affect and establishing an emotional bond (especially through increased mean pitch: Werker et al., 1994; Trainor et al., 2000; Singh et al., 2002); (c) aiding children in the task of language learning (especially through vowel hyper-articulation: Kemler Nelson et al., 1989; Singh et al., 2008; Song et al., 2010). Further investigation of vowel hyper-articulation has demonstrated that its application often relates to the speaker's perception of the listener's linguistic abilities (Burnham et al., 2010; Rice and Burnham, 2010; Lam and Kitamura, 2012); indeed, Xu et al. (2013) found that vowel space area in the speech of Australian English-speaking mothers increased from ADS to an unfamiliar adult to speech directed to a dog, to speech directed to a parrot with the ability to repeat speech, to IDS.

Burnham et al. (2002) and Uther et al. (2007) explored the possibility that a speaker's relationship with various types of interlocutors, including children and unfamiliar adults, but also foreign adults and even pets, could predict which types of special acoustic and prosodic features were applied. Burnham et al. (2002) showed that Australian English-speaking mothers addressed their pets and infants with similar degrees of increased pitch and affect, compared with when they addressed unfamiliar adults, but that only the mothers' speech to infants (not to pets or unfamiliar adults), featured vowel hyper-articulation (presumably a didactic feature of IDS). Uther et al. (2007) followed Burnham et al. (2002), but replaced pets with non-native English speakers. They showed that British English-speaking mothers addressed infants and foreigners with similar degrees of vowel hyper-articulation (presumably for didactic effect), but that speech to foreigners (FDS) lacked the increased mean pitch of IDS, indicating an absence of affective prosodic modification, relative to speech to native-speaker adults.

Indeed, since at least the 1970s, IDS and CDS have been compared with foreigner-directed speech styles (FDS: Ferguson, 1975; Freed, 1981a,b), and some authors even considered FDS to “derive” from IDS/CDS (DePaulo and Coleman, 1986). Similar acoustic and prosodic features have been claimed for FDS as for IDS/CDS, including longer vowel durations and/or slower speech rates, and vowel hyper-articulation. These features are also often claimed for a more general “clear speech” style that speakers may produce in noisy environments, when interacting with the hearing-impaired, when reading out loud, and when asked to enunciate clearly (Uchanski, 2005). FDS in both spoken and signed languages has further been described as involving fewer sandhi effects than speech to native-speaking adults (Henzl, 1979; Swisher, 1984; Tweissi, 1990). Gesture accompanying spoken French and Dutch FDS in Belgium has been shown to involve modifications, relative to gesture to native-speaking adults (Prové et al., 2022). But the acoustic and prosodic properties of FDS have not been studied comprehensively for all languages for which IDS/CDS data are available.

Comparisons of FDS with speech to native-speaking adults have shown slower FDS speech rates, sometimes with more pauses, for: English (Henzl, 1979; Ulichny, 1979; Warren-Leubecker and Bohannon, 1982; Wesche and Ready, 1985; Bobb et al., 2019; but see Arthur et al., 1980; Kühnert and Antolík, 2017), Czech (Henzl, 1973, 1979), French (Kühnert and Antolík, 2017, but see Wesche and Ready, 1985), German (Henzl, 1979), and Jordanian Arabic (Tweissi, 1990). Several studies found that English foreigner-directed speech (in the U.S., Scotland and England) featured vowel hyper-articulation, relative to speech directed to native-speaker adults (Knoll and Scharrer M., 2007; Scarborough et al., 2007; Uther et al., 2007; Knoll et al., 2009; Hazan et al., 2015; Bobb et al., 2019), but other studies on English did not yield this result (Knoll and Scharrer M. A., 2007; Knoll et al., 2015). In Norwegian (Sikveland, 2006) and Omani Arabic (Al-Kendi and Khattab, 2019; Al-Kendi, 2020, pp. 221–233), FDS has been found to feature increased first formant (F1) values for corner vowels, indicating a more open mouth and/or increased vocal effort (Ferguson and Kewley-Port, 2002). Exploration of pitch modifications in FDS compared with native-speaker ADS has yielded mixed results: from no modifications in British English FDS (Uther et al., 2007) to increased mean pitch in Omani Arabic FDS (Al-Kendi and Khattab, 2019).

Several studies have compared acoustic and prosodic features of IDS or CDS with those of FDS and native-speaker-directed ADS, resulting in mixed findings. In an early study in which Mandarin speakers simulated speech to babies, foreigners, and native-speaker adults, pitch contours in the simulated IDS and FDS differed (Papoušek and Hwang, 1991). Uther et al. (2007) showed that British FDS lacked the increased mean pitch, wider pitch range, and longer vowel durations evinced by British IDS relative to ADS, but that IDS and FDS had similar degrees of vowel hyper-articulation (also found by Kangatharan, 2015). Bobb et al. (2019) found that simulated IDS and FDS by speakers of American English showed significant differences in mean pitch (IDS > FDS), but not pitch range. Biersack et al. (2005) found that British English speakers produced longer vowels when addressing imaginary children, but longer pauses, rather than vowels, when addressing imaginary foreign adults, but this finding was not replicated by Uther et al. (2007), using naturalistic data. Knoll and Scharrer M. A. (2007) were unable to replicate the vowel hyper-articulation finding of Uther et al. (2007) using simulated IDS, ADS, and FDS interactions by undergraduate native speakers of British English, but did successfully replicate Uther et al.'s findings when the simulation was by actresses (Knoll et al., 2009, 2011). Lorge and Katsos (2019) compared simulated recipe explanations by English monolinguals and bilinguals directed to a 10-year-old child, a native English speaker, and a non-native English speaker; CDS to the 10-year-old child featured higher mean pitch, increased pitch range, and vowel hyper-articulation than ADS, while FDS featured a slower speech rate than ADS. Only the bilinguals also used vowel hyper-articulation in FDS.

IDS and CDS styles that differ lexically, acoustically, and/or prosodically from ADS styles are widespread and found in diverse communities around the world. But it has been claimed that some communities employ no special speech styles when addressing infants and young children; one of these purported exceptions is the Kaluli speech community of Papua New Guinea (Schieffelin, 1990). The languages of the New Guinea region constitute 20% of languages in the world today (Palmer, 2017), and therein, those of Papua New Guinea constitute at least 10% of languages in the world (Kik et al., 2021). If the absence of a special IDS or CDS style is an areal feature, a sizable proportion of the world's speech communities could lack such a style. Recent research into child language development in other communities of Papua New Guinea (listed in Hellwig et al., Forthcoming) has successfully identified various special qualities of IDS or CDS, including nursery lexicon (Aikhenvald, 2008; Stebbins, 2011; Sarvasy, 2017, 2019), consonant alteration (Schieffelin and San Roque, forthcoming; Rumsey, 2017; Sarvasy, 2019) and pitch modification (Frye aus Schwerte, 2019). To date, however, IDS or CDS in any language of Papua New Guinea has not been thoroughly analyzed acoustically, relative to conversational ADS in the same language (Sarvasy et al., 2019 is a pilot study on which the current study builds). Indeed, since Kaluli adults are said to maintain that children should hear only well-formed speech, to learn to speak correctly (Schieffelin, 1990), it could be the case that Kaluli adults, although possibly not employing modified lexical or other grammatical features in speech to children, do practice vowel hyper-articulation, to ensure that children are exposed to proper speech sounds.

Most of the literature on FDS, including its acoustic and prosodic features, targets major world languages, spoken in industrialized countries by literate communities. The notion that vowel hyper-articulation is a basic feature of FDS remains to be validated with data from small speech communities without traditions of literacy, where daily life may involve interactions only with known members of a small, closed community (*cf*. Wray and Grace, 2007). The languages of Papua New Guinea, which represent an outsized portion of the world's total, are overwhelmingly spoken by fewer than 10,000 speakers each, often in this type of closed community (Kik et al., 2021). If people rarely interact with non-native speakers of their language, one might assume that they might not employ a uniform FDS style.

In this study, we examine acoustic features of vowels in CDS, ADS, and speech that may be termed FDS, since it was directed at a non-native speaker (but could also be considered to exemplify a more general story-telling performative style), in the Towet dialect of the Papuan language Nungon, spoken by about 300 people in remote Towet village in the Uruwa River valley in NE Papua New Guinea (Sarvasy, 2017).

Previous work established that Nungon CDS involves several optional features that differentiate it from ADS: nursery lexicon, consonant alteration, and special morphosyntax (Sarvasy, 2019, 2020, 2021a). These features are evident in speech to infants and in speech to children of up to 3 years and even older. But the degree to which Nungon CDS vowels can be said to differ acoustically from ADS was not examined in previous studies, which also did not examine prosodic features of CDS, relative to ADS. Case studies of child acquisition of Nungon morphosyntax established that morphosyntactic complexity (verbal inflections, length of complex sentences, use of complex predicates) in the speech of two children increased substantially from about 3 years of age. By the same measures, morphosyntactic complexity in CDS to the same children also increased from when the children were about 3 years old (Sarvasy, 2019, 2020, 2021a). Further, a special Nungon morphosyntactic alteration found only in young children's speech, IDS, and CDS (not ADS) declines markedly in frequency in maternal CDS to children of about 3 years of age or more (Sarvasy, 2019). Nungon-speaking parents thus seem to exhibit “fine-tuning”—adjusting their own speech to the child's perceived cognitive and linguistic sophistication (Bohannon III and Marquis, 1977; Soderstrom, 2007)—in the morpho-syntactic domain. It is as yet unknown whether Nungon CDS to children under 3 years also features acoustic differences from Nungon CDS to children over 3 years.

Nungon speakers communicate in a world of intimates and classificatory relatives, without strangers (Wray and Grace, 2007). They rarely interact with non-native speakers of dialects of Nungon or of the closely related language Yau. A handful of people, mostly women, marry into the region from adjoining regions, where distantly related languages are spoken. Apart from the first author and the biologist Gabriel Porolak, no outsiders not married into local families have learned Nungon in at least the past two decades or so. Outsiders who travel fleetingly through the region usually speak the unrelated lingua franca Tok Pisin, which most Nungon speakers under about 40 understand and can speak.

Although true outsiders (people originating outside the Uruwa River valley) rarely learn Nungon, the linguistic situation among speakers of Nungon is complex. Each of the six Nungon-speaking villages has its own distinct dialect, with a particular accent, and which shares <90% of key vocabulary with other villages (Sarvasy, 2013, 2017). People marry into other villages, travel between them, and interact with speakers of other Nungon dialects on a regular basis. If Nungon has an identifiable FDS style, this could be rooted in modes of cross-dialect interaction, rather than norms for speaking Nungon to true foreigners (such as the first author, Gabriel Porolak, and the few in-married foreigners). Sarvasy (2017) also notes that there seem to be conventional ways to interact with Nungon speakers who have speech impediments or are intellectually disabled, which involve increased loudness of speech, increased use of conventionalized gestures, and possibly increased lip movements; this could represent another conventionalized Nungon clear speech style, potentially related to FDS.

We investigated vowel space size, vowel duration, mean pitch, and pitch range in Nungon CDS in the Towet village dialect to children aged 2;2–3;10. We compared the Nungon CDS acoustic results to results from dyadic adult Nungon conversations (conversational ADS) by the same speakers from the CDS data. We then compared these CDS and conversational ADS results to results for the same measures from Nungon monologues that had been recorded as semi-performances, with a non-native Nungon speaker (the first author) as primary listener (“MONO”; potential FDS). We aimed to evaluate the following, for the Towet village dialect of Nungon: (a) whether Nungon CDS vowels were hyper-articulated, relative to conversational ADS; (b) whether Nungon CDS featured increased mean pitch, expanded pitch range, longer vowel durations, and/or an enhanced contrast between phonologically short and phonologically long vowels, relative to ADS; and (c) how these acoustic measures patterned in the Nungon monologues with a non-native listener. Additionally, we evaluated (d) whether acoustic features of Nungon CDS vary with children's age, and whether (f) women and men differed in their use of CDS.

## Methods

Nungon has six phonemic vowels: an unrounded high front /i/, an unrounded mid front /e/, an unrounded low central /a/, a rounded high back /u/, a rounded mid-high back /o/ with extra lip protrusion, and a rounded mid-low back /*O*/ (Sarvasy, 2017; Sarvasy et al., 2020). Nungon is slightly unusual typologically in having more phonemic back vowels than front vowels. All the Nungon vowels can occur as either phonologically short or long; this is lexically determined. Details on vowel trajectories, using a multi-point analysis technique, are in Sarvasy et al. (2020).

We examined vowel tokens from three corpora for this study: CDS, ADS, and what we term MONO (monologual narratives directed at a non-native Nungon speaker with foreign appearance). We targeted the first two vowel formant values (F1 and F2), duration, and mean and range of fundamental frequency (F0: an objective measurement that is closely related to pitch).

### CDS dataset

Child-directed speech was extracted from a corpus of child-caregiver conversation from a longitudinal study of eight children acquiring Towet Nungon (Sarvasy, 2021c). This study occurred in two parts, with two different cohorts. The first cohort of five children were recorded for 1 h monthly for 2 years between 2015 and 2017. The second cohort of three children were recorded for 4 h within a single week each month over a 5-month period in 2019. Parents were paid an honorarium of 1,000–1,500 Papua New Guinean kina for their participation in the study, plus a gift, and research assistants were paid for each recording session and transcription they completed.

In each recording session, the target child sat with one or both parents and, sometimes, a sibling, in a natural indoor or outdoor setting. The parents understood that the purpose of the recording sessions was to elicit speech from the child in a relatively natural manner, to be able to study how the child's language development transpired. Parents and children usually looked at picture books together, or discussed events or activities the child had or would participate in, punctuated by occasional discussion of what they observed from their seated location (people walking by, etc.). The sessions were audio- and video-recorded by a local research assistant, usually the classificatory aunt or uncle of the child. Interaction was videorecorded using a Canon IXUS 190 digital camera held by the research assistant or mounted on a tripod. Interaction was audio-recorded with a Zoom H5 recorder mounted on a tripod and pointed toward the target child. Recordings were done at a 44.1 kHz sampling rate, in WAV format. Recordings were transcribed in Mid-CHAT format (MacWhinney, 2000) by native Towet Nungon speakers on Lenovo laptops in Towet village, using solar power.

Twelve recording sessions, involving six target children (three girls and three boys) and one or both of their parents, were used for analysis here. Ten sessions were selected according to whether their digital transcriptions, originally completed by Nungon speakers in the villages, had been finalized and checked; the other two were selected because the adult interlocutors had previously also recorded a monologue in the MONO dataset, to be analyzed here (see below). At the time of recording, three children's ages were in the range 2;2–2;9 (a girl, TO, recorded at ages 2;2 and 2;9; a girl, MA, recorded at age 2;7, and a boy, AB, recorded at age 2;7), and the other three were aged 3;0–3;10 (a girl, AR, recorded at age 3;10; a boy, NI, recorded at ages 3;0, 3;1, and 3;2; and a boy, DA, recorded at ages 3;5, 3;6, 3;7, and 3;8). The adults whose speech was analyzed here were all in their twenties or thirties at the time of the recordings, and all were native speakers of the Towet Nungon dialect.

### Dyadic adult conversational dataset (ADS)

We commissioned six 15-min recordings of free conversation between two Towet Nungon-speaking adults each, specifying that the dyads should include parents from the CDS dataset. In the sessions, two adult Nungon speakers, usually classificatory relatives, sat close to each other and spoke about shared past experiences, or related anecdotes from their separate experiences. Note that because of the nature of these small communities (Wray and Grace, 2007), it is impossible to find speakers of the same Nungon dialect (of which there are 100-350 speakers) who are not classificatorily related to each other and do not know each other, so the usual practice in studies comparing CDS with a “baseline” ADS in which an adult addresses an unfamiliar adult was impossible to achieve here. The sessions were recorded using a Zoom H5 recorder mounted on a tripod, placed between the two speakers. Recordings were done at a 44.1 kHz sampling rate, in WAV format. All participants received an honorarium of 50 Papua New Guinean kina each, and the assistants who ran the recording sessions were also compensated for their time. All speakers but one who were recorded conversing here also feature in the CDS sample. The only speaker who does not also feature in the CDS dataset, DI, was in her thirties at the time of the recording.

### Adult performed monologues with non-native listener (MONO)

The final dataset from which vowel tokens were extracted involved monologual narratives that adult Towet Nungon speakers recorded individually between September 2011 and March 2013, with the first author, a non-native speaker of Nungon, as primary listener (wearing headphones and usually holding a digital recorder in one hand). The first author's fieldwork was performed monolingually (Sarvasy, 2016), meaning that she always spoke only Nungon to Nungon speakers, without recourse to a contact language such as the Papua New Guinean lingua franca Tok Pisin or English. Nungon speakers would thus have observed her language development over a series of field trips (generally 1.5–2.5 months in length), from very early stages in mid-2011 to near-fluency in 2013. The recordings used here were made at different stages of the author's linguistic development, but even for the earlier recordings, the author was fluent enough to be able to discuss the recording context and protocols with each speaker herself. Elsewhere, Sarvasy (2021b) notes that the degree of intimacy she had with a speaker could be a factor in determining the length and amount of detail in a recorded monologue.

The speakers usually framed each recording as a *hat* “story,” and most had approached the first author in advance of the recording session to notify her that they intended to record one or more stories for her; the topics were either chosen by the speakers in this way, or were responses to questions from the first author regarding, for instance, life in the olden days. Speakers were aware that these recordings would support the first author's research into the Nungon language grammar (Sarvasy, 2017). They had further been warned by the local government Councillor, early in the first author's research into the grammar, that he expected community members to provide the author with only *maa orogo* “good language,” by which he intended that *maa muyam* “cursing” should not be used, but which highlights a type of pressure some speakers might have felt to produce “good” speech.

Although we could categorize these recordings as FDS, simply because of the identity of the interlocutor, we will refer to them here as MONO because at least two other factors in the recording context could have led speakers to produce speech differently than in normal conversation. First, with the recording's purpose in mind, speakers could have aimed to speak clearly to produce a clear record for posterity. Second, the performative aspect of the recording context, in which speakers entered a hut with the express purpose of telling a narrative well, could have led to extra care in speech: they were conscious that they were performing a storytelling act, for audiences (present and future). Future work may attempt to separate these effects from the effect of non-native interlocutor in a controlled way, but we are unable to do this definitively with the present data.

Of a corpus of 221 such recorded and transcribed texts (Sarvasy, 2015), 15 were analyzed for the present study. All of the monologues analyzed were narratives describing personal experiences of the speakers, except for two local legends, and a personal introduction, where the speaker described her family origin. These were recorded in close range using the built-in twin microphones of a Zoom H4N Handy audio recorder with no external microphone, at a 44.1 kHz sampling rate, in WAV format. Speakers were paid 10–20 Papua New Guinean kina for a storytelling session. These were digitally transcribed by the first author in close consultation with each speaker him- or herself in Towet village in 2011–2013.

### Selection of tokens and acoustic analysis

In each dataset, transcriptions and/or audio recordings were searched for words that included the six phonemic Nungon vowels in word-initial, word-medial and word-final environments, and not adjacent to nasals (to avoid coarticulation and prosodic effects). While the ideal was to find the same words (e.g., *agep* “firm” for the vowels /a/ and /e/) in all corpora and uttered by all speakers, the uncontrolled nature of the corpora made this difficult, so the words extracted varied slightly from speaker to speaker. If tokens of a word exceeded 20 in a single session by one speaker, only the first 20 tokens were used. The corresponding audio was hand-screened for obviously poor recording quality and obscuring background noise. Tokens of all six vowels were used to examine mean pitch and pitch range, and vowel duration; tokens of the corner vowels /i/, /a/, and /u/ were used to determine vowel space size, following the method in García-Sierra et al. (2021).

For the CDS dataset, we selected adult vowel tokens from the 12 sample child-caregiver interaction recordings, coded according to the identity and sex of the adult. In analyses, we do not differentiate between parents and the three Nungon-speaking assistants who occasionally interact with the children in the recording sessions (LY, JA and ST in Table 1). The reason for this is that the assistants were classificatory close relatives of the children and habitually interacted with them, occasionally caring for them, in the close-knit, 30-household, Towet village community, where child-rearing has a communal character. In selecting vowel tokens, we avoided tokens that were adjacent to nasal segments, since Sarvasy et al. (2020) showed that coarticulation effects are present in Nungon vowels adjacent to nasals. We also tried to extract vowels from a fixed set of words, as much as possible, for all speakers. For instance, the word *agep* “firm, loud” occurs frequently in these recordings, addressed to the target child, when the parent wants the child to speak louder. This was a good source for vowel tokens of /a/ and /e/. Table 1 shows the CDS vowel tokens used. As mentioned above, Nungon distinguishes lexically determined phonological vowel length contrasts, such that each vowel takes both a phonologically short and long form. These length contrasts are not shown in Tables 1–**3**.

**Table 1:** Number of tokens for each vowel, by speaker, in the CDS dataset.

**Speaker**	**Gender**	**Age of child**	**/i/**	**/e/**	**/a/**	**/*O*/**	**/o/**	**/u/**
YI	Female	3;5–3;8	29	37	66	53	22	21
LY	Female	3;5–3;10	5	5	32	13	9	11
NU	Female	3;1–3;2	11	44	76	100	24	30
AM	Female	3;10	7	33	37	10	8	15
TM	Female	2;2, 2;9	74	52	92	76	14	25
DE	Male	2;7	17	25	49	33	6	19
BO	Male	2;7	8	11	24	21	2	16
MA	Male	2;9	14	8	20	33	6	7
JA	Male	3;5–3;6	7	6	29	51	6	1
ST	Male	3;0–3;10	11	17	50	33	4	15
Total			183	238	475	423	101	160

Table 1 shows that one woman and three men addressed the 2-year-olds (2;2–2;9), while four women and two men addressed 3-year-olds (3;0–3;10). We will compare features of CDS to these two different age groups in the Results.

For the ADS dataset, sections of the conversations were transcribed by the first author. From these sections, we extracted a subset of vowel tokens not adjacent to nasal consonants for analysis. Table 2 gives the number of vowel tokens per speaker used from the conversational ADS dataset here.

**Table 2:** Number of tokens for each vowel, by speaker, in the conversational ADS dataset.

**Speaker**	**Gender**	**/i/**	**/e/**	**/a/**	**/*O*/**	**/o/**	**/u/**
YI^*^	Female	21	19	50	85	19	13
LY^*^	Female	23	25	53	45	10	15
NU^*^	Female	12	15	29	23	8	4
DI	Female	9	6	9	18	10	2
DE^*^	Male	1	0	7	1	1	4
BO^*^	Male	5	5	6	7	4	2
MA^*^	Male	11	9	14	20	7	9
JA^*^	Male	10	14	22	22	3	11
Total		92	93	190	221	62	60

* *Indicates speakers who also appear in the CDS dataset*.

Finally, for the MONO dataset, vowel tokens not adjacent to nasal segments were extracted. Five of the speakers in the MONO dataset also feature in the CDS and ADS corpora. Of the remaining three, one, NK, was in her twenties at the time of the recording, one, RO, was in her forties, and the third, OR, was in her late forties or early fifties.

Comparison of Tables 1–3 shows that, while eight of nine ADS speakers also feature in the CDS dataset, only five MONO speakers feature in the CDS dataset. Of these, one (ST) does not feature in the conversational ADS dataset, yielding just two male and two female speakers who feature in all three corpora. For this reason, we performed three sets of analyses: one using just tokens from the four speakers who featured in all three datasets (comparison of CDS, ADS, and MONO); one using tokens from the seven speakers who featured in both CDS and ADS datasets (comparison of just CDS and ADS); and one with all 14 speakers (comparison of CDS, ADS, and MONO); results will be summarized for the three groups separately in **Table 8**.

**Table 3:** Number of tokens for each vowel, by speaker, in the MONO dataset.

**Speaker**	**Gender**	**/i/**	**/e/**	**/a/**	**/*O*/**	**/o/**	**/u/**
YI^*^	Female	24	24	45	44	14	16
LY^*^	Female	2	25	8	47	1	7
NK	Female	51	8	50	61	30	26
OR	Female	9	22	28	62	13	3
RO	Female	51	12	88	73	47	41
DE^*^	Male	5	5	15	18	1	8
BO^*^	Male	39	26	45	118	31	14
ST^*^	Male	29	53	46	75	17	30
Total		210	175	325	498	154	145

* *Indicates speakers who also appear in the CDS dataset*.

For CDS and MONO, existing transcriptions were manually aligned at utterance level before the use of Munich Automatic Segmentation System (MAUS) for forced alignment at the word and phonetic level. WebMAUS (Kisler et al., 2017) was used following two steps: grapheme-to-phoneme conversion, followed by alignment in the “language independent” mode that does not require language training in advance, which seems to be a good option for under-described languages (Kisler et al., 2012; Jones et al., 2019). Subsequently, the segmentation by MAUS at the phonetic level was manually checked and adjusted, as there were a large number of cases in which misalignment occurred. For conversational ADS, as described above, there were no existing transcriptions, so vowels were segmented directly by hand by the first author.

The vowels' duration, F0, and formant values were extracted using the analysis techniques of previous similar studies (e.g., Williams and Escudero, 2014; Kashima et al., 2016; Sarvasy et al., 2020): 30 evenly distributed points starting from the 20% point to the 80% point of the vowel duration in Praat (Boersma and Weenink, 2021). We excluded the initial and final 20% of the vowel token in order to minimize coarticulation influences (Williams and Escudero, 2014). Following the method described in Williams and Escudero (2014), we then smoothed the series of formant values (or trajectories) for each vowel to reduce the influence of noisy formants by fitting each vowel token with parametric curves, specifically, the discrete cosine transform (DCT) in MATLAB. Then the vowel duration and formants values were averaged over the speakers and across the different positions. The pitch range was calculated by finding the maximum and minimum F0 values among the 30 evenly distributed points. Linear mixed modeling and *post-hoc* analysis were performed in R using the lmerTest and emmeans packages (Searle et al., 1980; Kuznetsova et al., 2017).

## Results

We first present a comparison of acoustic characteristics of vowels in CDS addressed to the two age-divided groups of children, three children per group (2;2–2;9 vs. 3;0–3;10). We found that the only acoustic feature that differed significantly in CDS to the younger and older cohorts was vowel duration. Thus, in the remaining analyses, we compare vowel formants, mean pitch, and pitch range, for CDS to all children, but the two age cohorts are separated when examining vowel duration and duration contrasts. We then compare the acoustic characteristics of vowels in Nungon CDS with those of conversational ADS and monologue narrative (MONO).

### CDS to children of different age ranges compared

Tables 4, 5 give an overview of vowel token numbers, first and second formant values, durations, mean pitch values, and pitch ranges, in CDS to the two age cohorts of children: “2-year-olds” (2;2–2;9), and “3-year-olds” (3;0–3;10).

**Table 4:** Number of tokens and formant values for the six Nungon vowels in CDS.

			**/i/**	**/e/**	**/a/**	**/*O*/**	**/o/**	**/u/**
2-year-olds	F	*N*	52	119	211	176	63	77
		F1	440 (160) 22	443 (87) 8	764 (220) 15	525 (134) 10	512 (155) 20	424 (98) 11
		F2	2,133 (379) 22	2,177 (255) 8	1,797 (158) 15	1,465 (381) 10	1,263 (360) 20	1,617 (324) 11
	M	*N*	18	23	79	84	10	16
		F1	351 (87) 21	453 (114) 24	590 (156) 18	529 (148) 16	478 (79) 25	414 (88) 22
		F2	1,976 (203) 21	1,814 (304) 24	1,559 (234) 18	1,378 (291) 16	1,209 (261) 25	1,432 (388) 22
3-year-olds	F	*N*	52	119	211	176	63	77
		F1	440 (160) 22	443 (87) 8	764 (220) 15	525 (134) 10	512 (155) 20	424 (98) 11
		F2	2,133 (379) 22	2,177 (255) 8	1,797 (158) 15	1,465 (381) 10	1,263 (360) 20	1,617 (324) 11
	M	*N*	18	23	79	84	10	16
		F1	351 (87) 21	453 (114) 24	590 (156) 18	529 (148) 16	478 (79) 25	414 (88) 22
		F2	1,976 (203) 21	1,814 (304) 24	1,559 (234) 18	1,378 (291) 16	1,209 (261) 25	1,432 (388) 22

*Formant values (Hz) averaged for the six vowels produced by 10 speakers: mean (standard deviation) standard error*.

**Table 5:** Duration (ms), mean F0, and F0 range (Hz) for vowels in Nungon CDS by 10 speakers: mean (standard deviation) standard error.

		**CDS to 3-year-olds**	**CDS to 2-year-olds**
Long	*N*	69	81
	Duration	128 (46) 6	158 (173) 19
Short	*N*	859	571
	Duration	73 (47) 2	114 (105) 4
Female	*N*	698	333
	Mean F0	220 (55) 2	207 (63) 3
	F0 range	18 (21) 2	18 (23) 3
Male	*N*	230	319
	Mean F0	140 (41) 3	130 (38) 2
	F0 range	10 (22) 3	9 (10) 2

### Vowel triangles in CDS

**Figure 1:**
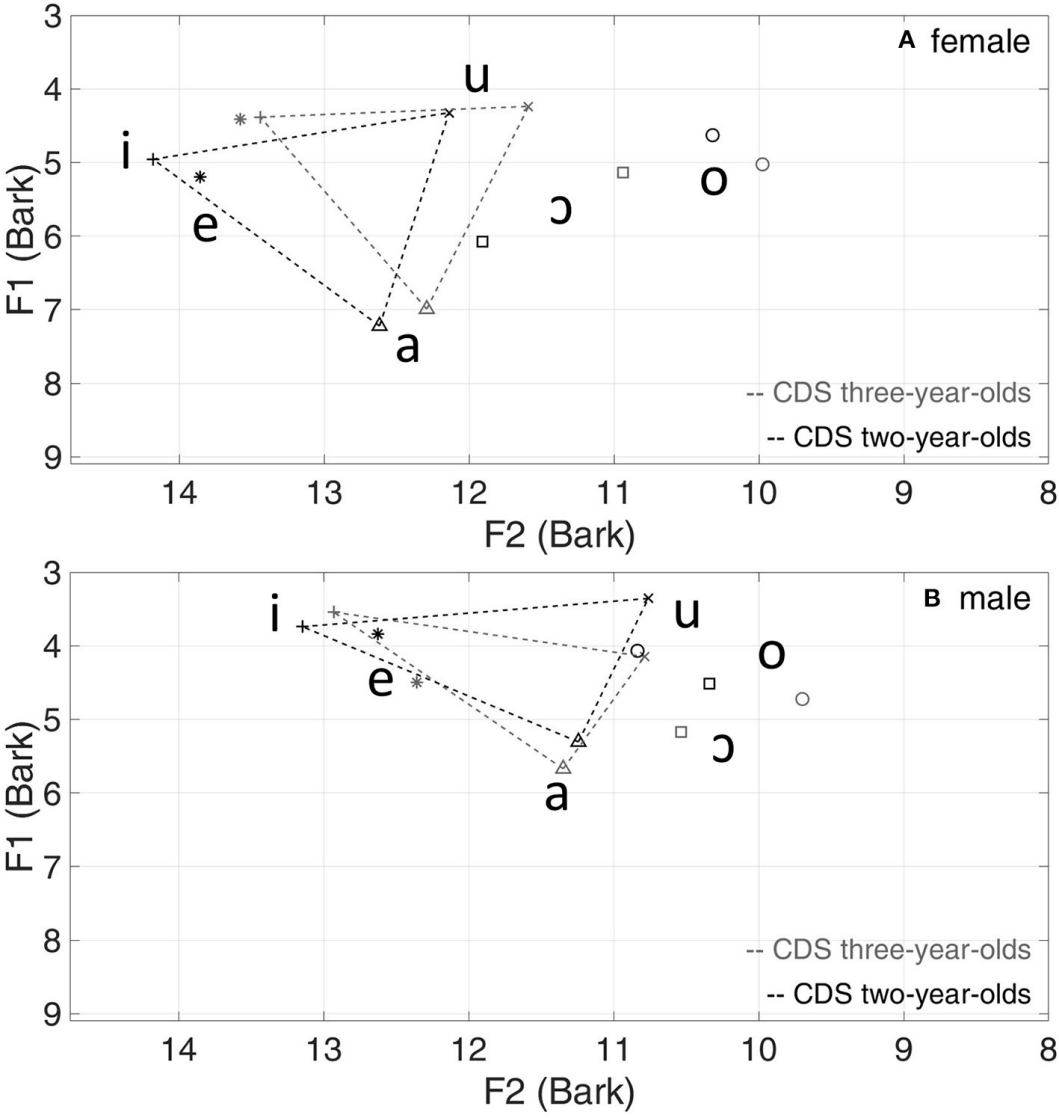
Mean vowel formants (Bark) and triangles of the 2-year-olds vs. 3-year-olds in CDS for women **(A)** and men **(B)**.

Figure 1 presents the vowel triangles from data for the 10 speakers in the CDS dataset. For women, a shift of the vowel triangle toward the bottom left (i.e., increase of both F1 and F2) can be observed for the 2-year-olds, compared with the 3-year-olds. Recall, however, from Table 1, that only one woman features in the 2-year-olds group, compared with four women in the 3-year-olds group. For men, there is little change in the placement of the vowel triangle relating to age of the children.

From the vowel triangle plots, the vowel space area (VSA) was then derived based on the vowels /i/, /a/, and /u/, following the method used by García-Sierra et al. (2021), for further comparison between CDS to the 2-year-olds and CDS to the 3-year-olds. A linear mixed model was run with VSA as a dependent variable, child's age (2- and 3-year-olds) and speaker gender (women and men) as independent variables, and speaker as a random intercept. There was no significant interaction between child's age and gender [*F*_(1,8)_ = 0.09, *p* = 0.78]. The difference in VSA between men and women was also not significant for both younger (*p* = 0.97; *N* = 4) and older groups (*p* = 0.67; *N* = 6), suggesting that men and women could be collapsed for analysis. Thus, a simpler linear model with child's age (2- and 3-year-olds) as one independent variable was used. *Post-hoc* analysis showed that VSA for CDS to 2-year-olds (Mean = 0.084 LnHz^2^, SE = 0.020) was not significantly different from that for CDS to 3-year-olds (Mean = 0.072 LnHz^2^, SE = 0.009, *p* = 0.54; *N* = 10), indicating no hyper- or hypo-articulation in CDS to the 2-year-olds compared with CDS to the 3-year-olds, for both women and men.

### Pitch mean and range in CDS

Linear mixed effects models were run with mean F0 and F0 range as dependent variables, child's age (2- and 3-year-olds) and gender (women and men) as fixed effects, and speaker as a random intercept. For mean F0, there was no significant interaction between child's age and gender [*F*_(1,1578)_ = 0.0006, *p* = 0.98]. As would be expected based on physical differences, women's mean F0 was higher than that of men ($\hat{\beta }$ = 70.8, *p* = 0.003). Pairwise comparisons showed no significant difference between mean F0 in CDS to 2-year-olds vs. CDS to 3-year-olds for women (*p* = 0.61; *N* = 1,031) and men (*p* = 0.56; *N* = 549). For F0 range, there was no significant interaction between child's age and gender [*F*_(1,1, 578)_ = 0.475, *p* = 0.52]. Women showed a larger F0 range than men ($\hat{\beta }$ = 7.56, *p* = 0.003), but neither sex showed a significant difference between F0 range in CDS to 2-year-olds and F0 range in CDS to 3-year-olds.

### Vowel durations and duration contrast in CDS

Linear mixed effects models were run with vowel duration as a dependent variable, vowel length (long and short) and child's age (2- and 3-year-olds) as fixed effects, and speaker as a random intercept. *Post-hoc* analysis showed no significant interaction between child's age and vowel length [*F*_(1,1578)_ = 0.388, *p* = 0.53]. Pairwise comparisons showed that the duration contrast between long and short vowels was significant in both CDS to 2-year-olds ($\hat{\beta }$ = 46.0, *p* < 0.001) and CDS to 3-year-olds ($\hat{\beta }$ = 54.9, *p* < 0.001), demonstrating that in neither variety is the usual Nungon phonological duration contrast minimized or lost. The duration of short vowels in CDS to 2-year-olds was significantly longer than that in CDS to 3-year-olds ($\hat{\beta }$ = 43.1, *p* < 0.001), and the same was found for long vowels ($\hat{\beta }$ = 34.2, *p* = 0.02). In sum, vowels in CDS to the 2-year-olds were found to last significantly longer than vowels in CDS to the 3-year-olds.

### CDS, ADS, and narrative monologues compared

Vowel token numbers, first and second formant values, durations, mean pitch, and pitch range for the ADS and MONO datasets are summarized in Tables 6, 7.

**Table 6:** Vowel token data for Nungon ADS and MONO.

			**/i/**	**/e/**	**/a/**	**/*O*/**	**/o/**	**/u/**
ADS	F	*N*	65	65	141	171	47	34
		F1	428 (54) 7	499 (52) 6	789 (139) 12	595 (80) 6	549 (71) 10	439 (56) 10
		F2	2,313 (306) 7	2,194 (303) 6	1,743 (171) 12	1,440 (274) 6	1,201 (356) 10	1,639 (266) 10
	M	*N*	27	28	49	50	15	26
		F1	343 (48) 9	413 (64) 12	576 (94) 13	488 (55) 8	475 (68) 18	347 (42) 8
		F2	2,058 (229) 9	2,069 (111) 12	1,581 (191) 13	1,359 (292) 8	1,136 (256) 18	1,378 (376) 8
MONO	F	*N*	137	91	219	287	105	93
		F1	411 (60) 5	537 (76) 8	907 (191) 13	684 (106) 6	564 (107) 10	442 (79) 8
		F2	2,381 (268) 5	2,235 (182) 8	1,737 (133) 13	1,332 (186) 6	1,039 (230) 10	1,402 (388) 8
	M	*N*	73	84	106	211	49	52
		F1	338 (48) 6	444 (52) 6	656 (87) 8	522 (62) 4	432 (42) 6	332 (38) 5
		F2	2,242 (251) 6	2,038 (286) 6	1,486 (156) 8	1,146 (179) 4	927 (196) 6	1,177 (383) 5

*Number of tokens and formant values (Hz) averaged for the six vowels: mean (standard deviation) standard error*.

**Table 7:** Duration (ms), mean F0 and F0 range (Hz) for vowels in Nungon ADS and MONO: mean (standard deviation) standard error.

		**ADS**	**MONO**
Long	*N*	76	153
	Duration	134 (44) 5	175 (79) 6
Short	*N*	642	1,354
	Duration	82 (39) 2	89 (46) 1
Female	*N*	523	932
	Mean F0	201 (32) 1	213 (38) 1
	F0 range	10 (12) 1	13 (16) 1
Male	*N*	195	575
	Mean F0	117 (14) 1	123 (16) 1
	F0 range	5 (4) 1	5 (7) 1

We ran three sets of analyses to compare CDS with the two types of adult-directed speech: conversational (the “ADS” dataset) and performative storytelling, directed at a non-native speaker (the “MONO” dataset). The ideal here would be to have obtained data for all three speech contexts from each speaker, such that all comparisons would be within-speaker. Unfortunately, our datasets include only four speakers who produced CDS, ADS and MONO (allowing for true within-speaker comparisons across those three contexts), and seven who produced both CDS and ADS (allowing for within-speaker comparisons across those two contexts). We thus also ran a further analysis that included all 14 speakers who feature in at least one of these three recording contexts, and compared the results with those of the smaller, within-speaker, analyses. That is: First, we compared only tokens from the four speakers who featured in CDS, ADS, and MONO; then we compared only tokens from the seven speakers who featured in both CDS and ADS, comparing those two datasets; then we compared tokens from all 14 speakers, comparing CDS, ADS, and MONO. The results from these three sets of analyses are in Table 8.

**Table 8:** Comparisons of vowel space area (VSA), F0 mean and range, and vowel duration for CDS, ADS and MONO dataset.

		**All 14 speakers**	**7 overlapping speakers in CDS and ADS**	**4 overlapping speakers in CDS, ADS and MONO**
VSA		MONO > ADS ~ CDS	ADS ~ CDS	MONO > ADS ~ CDS
Mean F0	F	MONO > CDS > ADS	CDS > ADS	MONO > CDS > ADS
	M	CDS > ADS > MONO	CDS > ADS	CDS > MONO > ADS
F0 range	F	CDS > MONO > ADS	CDS > ADS	MONO ~ CDS > ADS
	M	CDS > ADS ~ MONO	CDS > ADS	CDS ~ ADS ~ MONO (CDS > MONO)
Duration	Short	CDS_2-year-olds > MONO > ADS ~ CDS_3-year-olds	CDS_2-year-olds > ADS ~ CDS_3-year-olds	CDS_2-year-olds > MONO > ADS > CDS_3-year-olds
	Long	MONO ~ CDS_2-year-olds > ADS ~ CDS_3-year-olds	CDS_2-year-olds > ADS ~ CDS_3-year-olds	CDS_2-year-olds > MONO > ADS ~ CDS_3-year-olds

*Symbols ‘>’ and ‘~’ stand for ‘higher/larger than’ with and without significance, respectively*.

Overall, the results for smaller, within-speaker analyses are similar to those for the entire 14-speaker group, so the results from that group are the ones presented in depth in the following sections.

#### Vowel triangles in CDS, ADS, and MONO

**Figure 2:**
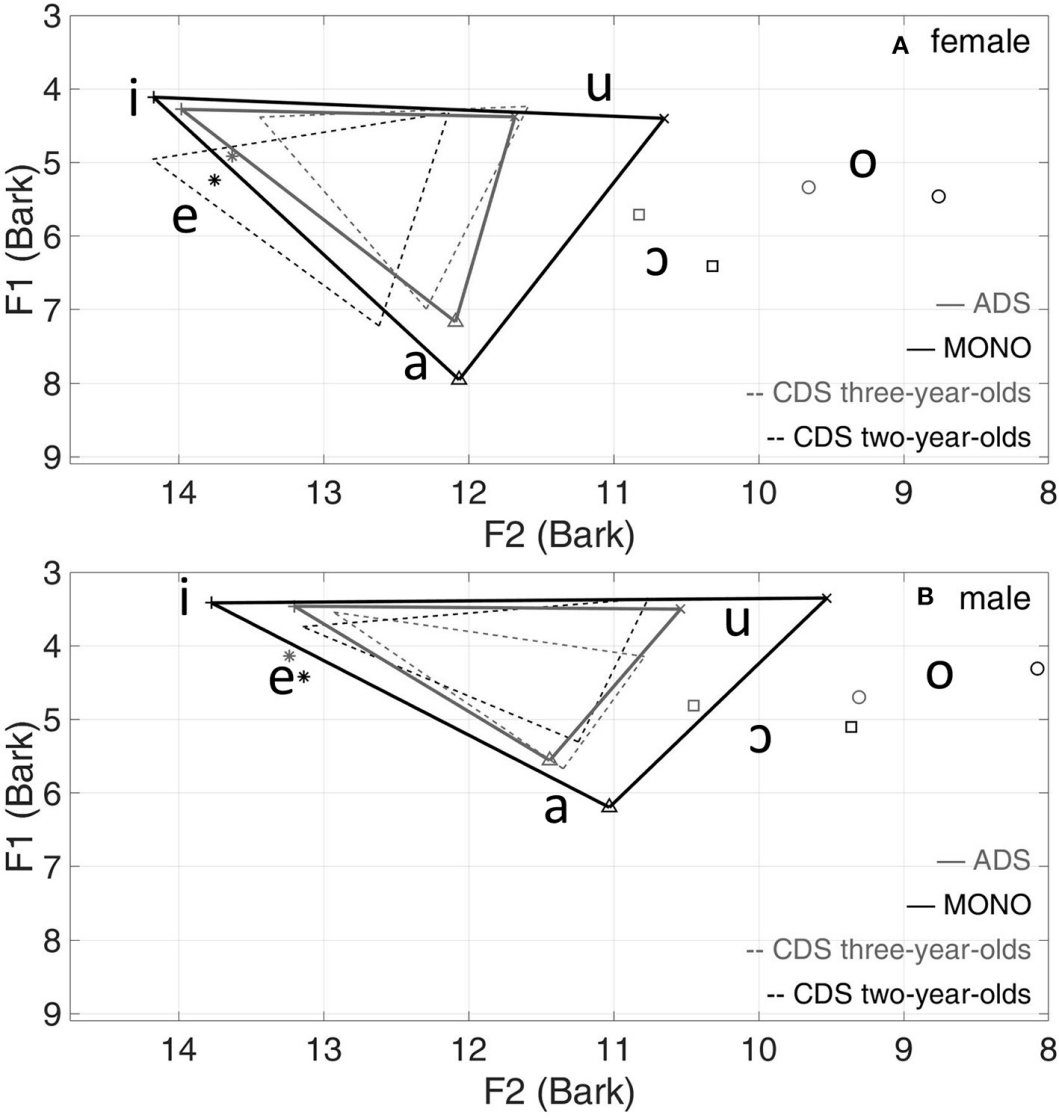
Mean vowel formants (Bark) and triangles of all speakers in CDS, ADS and MONO for women **(A)** and men **(B)**.

Figure 2 presents the vowel triangles from data for all 14 speakers in CDS, ADS and MONO corpora. MONO speech involves by far the largest vowel space, evaluated in terms of F1 and F2 of the three corner vowels. For both women and men, the vowel triangle for CDS to 3-year-olds is similar to that for ADS.

Since there was no significant difference in VSA between the CDS to 2-year-olds and CDS to 3-year-olds, the two groups were collapsed as “CDS” for the following comparison with ADS and MONO. There was no significant interaction between child's age and gender [*F*_(2,23)_ = 0.22, *p* = 0.80]. The difference in VSA between men and women was not significant for all three corpora (CDS: *p* = 0.75, *N* = 10; ADS: *p* = 0.55, *N* = 8; MONO: *p* = 0.85, *N* = 8), suggesting that men and women could be collapsed when the three corpora were compared. A linear model with dataset (CDS, ADS and MONO) as one independent variable showed that MONO has the largest VSA value (Mean = 0.236 LnHz^2^, SE = 0.029), significantly larger than ADS (Mean = 0.105 LnHz^2^, SE = 0.016; $\hat{\beta }$ = 0.131, *p* < 0.001) and CDS (Mean = 0.076 LnHz^2^, SE = 0.009; $\hat{\beta }$ = 0.159, *p* < 0.001), but the difference between ADS and CDS was not significant (*p* = 0.53; *N* = 18). The results for analyses including only overlapping speakers (those who feature in all three datasets) were similar to those with all speakers, as seen in Table 8.

### Pitch mean and range in CDS, ADS, and MONO

Since there was no significant difference in either mean pitch or pitch range between CDS to 2-year-olds and CDS to 3-year-olds, the two groups were collapsed as “CDS” in the following pitch analysis. Linear mixed effects models similar to those in 3.1.2 were used for pairwise comparisons of the three corpora. For mean F0, there was a significant interaction between dataset and gender [*F*_(2,3125)_ = 102.1, *p* < 0.001], and the trend for different corpora varied with gender. For women, F0 mean for MONO was significantly higher than that for CDS ($\hat{\beta }$ = 28.7, *p* < 0.001) and ADS ($\hat{\beta }$ = 52.4, *p* < 0.001). For men, CDS showed the highest mean F0, followed by ADS ($\hat{\beta }$ = 11.5, *p* = 0.002) and MONO ($\hat{\beta }$ = 22.6, *p* < 0.001). For F0 range, there was also a significant interaction between dataset and gender [*F*_(2,3125)_ = 8.1, *p* < 0.001]. For women, F0 range for CDS was the largest, but not significantly different from MONO (*p* = 0.12; *N* = 1,576); both CDS and MONO were significantly larger than ADS ($\hat{\beta }$ = 8.0, *p* < 0.001 for CDS and $\hat{\beta }$ = 5.8, *p* < 0.001 for MONO). For men, F0 range for CDS was also the largest, and significantly different from ADS ($\hat{\beta }$ = 3.3, *p* < 0.001) and MONO ($\hat{\beta }$ = 4.8, *p* < 0.001).

There were seven speakers (three women and four men) who featured in both the CDS and ADS corpora. As shown in Table 8, the results for these overlapping speakers were consistent with those for all speakers: for both women and men, mean F0 for CDS was significantly higher than that for ADS, and F0 range for CDS was also significantly larger than that for ADS. Results for the four overlapping speakers (two women and two men) featuring in all three corpora were also consistent with those for all speakers in general.

### Vowel durations and duration contrast in CDS, ADS, and MONO

Both phonologically short and long vowels in CDS to 2-year-olds were significantly longer in duration than those in CDS to 3-year-olds. Thus, for comparison of duration with ADS and MONO, CDS to 2-year-olds and CDS to 3-year-olds were separated. Short vowels in CDS to 2-year-olds were significantly longer than those in MONO ($\hat{\beta }$ = 23.4, *p* < 0.001), ADS, and CDS to 3-year-olds; those in MONO were significantly longer than those in ADS ($\hat{\beta }$ = 24.9, *p* < 0.001) and CDS to 3-year-olds, but those in ADS were similar to those in CDS to 3-year-olds (*p* = 0.06; *N* = 1,501). Long vowels in MONO were similar to those in CDS to 2-year-olds (*p* = 0.50; *N* = 234); those in CDS to 2-year-olds were significantly longer than those in ADS ($\hat{\beta }$ = 45.0, *p* < 0.001) and CDS to 3-year-olds, but those in ADS were similar to those in CDS to 3-year-olds (*p* = 0.98, *N* = 145). So for both short and long vowels, duration was exaggerated in CDS to 2-year-olds and in MONO compared with ADS, while duration in CDS to 3-year-olds was similar to that in ADS. In general, these results were consistent with those for overlapping speakers.

The duration contrast between phonologically long and short vowels was significant in all four corpora ($\hat{\beta }$ > 46.8, *p* < 0.001). For overlapping speakers (both across ADS, CDS and across ADS, CDS and MONO), the contrast remained significant ($\hat{\beta }$ > 46.3, *p* ≤ 0.001). That is, in none of these genres is the duration contrast between phonologically short and long vowels neutralized.

## Discussion

In this paper, we present a first study comparing acoustic and prosodic properties of vowels in CDS (to children aged 2;2-3;10), ADS (to familiar adults), and monologual speech directed at a foreigner, for the Towet dialect of the Papuan language Nungon, spoken by about 300 people in a remote mountain village of Papua New Guinea (of the 1,000 speakers of Nungon across all dialects). Our results show that Nungon CDS does not feature expansion of the vowel space, compared to conversational Nungon ADS. The Nungon CDS vowel triangle area is neither significantly smaller nor larger than that of conversational ADS. As seen in Table 8, this result applies for both women and men, and holds true when analyzed for three different cross-sections of our dataset: first, the seven speakers who produced both CDS and conversational ADS; second, the four speakers who produced CDS, conversational ADS, and narrative monologues (MONO); and third, the total 14 speakers who feature in at least one cross-section of our dataset: CDS, conversational ADS, and/or MONO. The size of the vowel triangle does not differ significantly for either men or women in CDS to children aged 2;2–2;9, compared with CDS to children aged 3;0–3;10. Since we did not examine data on Nungon vowel acoustics in IDS to infants under 12 months, or CDS to children under 2 years of age, we cannot rule out the possibility that Nungon IDS or CDS to children aged 0–25 months does indeed involve vowel hyper-articulation.

While we found that Nungon CDS to the children in our sample did not exhibit vowel hyper-articulation, Nungon MONO (our measure of FDS in Nungon, from monologual narratives told to a non-native listener holding a recording device) did. This pattern holds both for the four individual speakers who produced tokens of all three speech varieties (CDS, conversational ADS, and MONO), and for the vowel tokens of the entire group of 14 speakers across the three datasets. In terms of vowel space area, then, the Nungon pattern could be similar to that suggested by studies of Norwegian IDS (Englund and Behne, 2005, 2006; Englund, 2018) and CDS (Steen and Englund, 2022), and Norwegian FDS (Sikveland, 2006). Norwegian IDS and CDS (to children in “kindergartens,” 10–34 months of age) do not show vowel hyper-articulation relative to ADS, but Norwegians addressing second-language learners do display expansion of the vowel space (perhaps, in part, due to an overall more open mouth during speech).

Nungon-speaking adults, both male and female, used higher mean F0 and expanded F0 range in CDS than in ADS, even though the ADS samples here were conversations between adults who had known each other very well for many years, rather than strangers. Male caregivers have been shown to differ from female caregivers in pitch modifications in American English CDS (Warren-Leubecker and Bohannon, 1984, although see Fernald et al., 1989, where both men and women used increased mean pitch and expanded pitch range in American English IDS). At least in speech addressed to children aged 2;2–3;10, we found no such differences between Nungon-speaking women and men. Women and men both used higher mean F0 in Nungon CDS than in conversational ADS, and both used greater F0 range in CDS than in conversational ADS, as found for mothers and fathers speaking a range of major world languages by Fernald et al. (1989).

Women and men do differ in mean F0 and F0 range within the MONO dataset, and in the relationship between F0 in the MONO dataset and in the other two datasets. For women, MONO features the highest mean F0, followed by CDS, then ADS; men's highest mean F0 occurs in CDS, followed by MONO, then ADS. Women have similar F0 ranges in CDS and MONO, which are both higher than the F0 range in ADS. In contrast, men have a greater F0 range in CDS than in MONO and ADS, which have similar F0 ranges. This implies that men's performative FDS monologue style is similar in pitch features to their ADS, albeit with slightly higher pitch overall, while CDS is the most divergent for both pitch mean and range. For women, in contrast, the performative FDS monologue style is the outlier, rather than CDS; MONO exceeds CDS in mean pitch and pitch range. Previous studies have yielded mixed results in terms of increased mean pitch or pitch range in FDS, relative to native-speaker-directed speech; increased mean pitch was found for Omani Arabic FDS (Al-Kendi and Khattab, 2019) and for English FDS by Knoll and Scharrer M. A. (2007), but not by Biersack et al. (2005) or Uther et al. (2007). Increased pitch range has been found in Mandarin FDS (Papoušek and Hwang, 1991) and French FDS (Smith, 2007).

In sum, when Nungon CDS is compared to conversational ADS, women and men show very similar patterns of mean F0 and F0 range. For both women and men, Nungon CDS to children aged 2;2–3;10 features significantly higher mean F0 and greater F0 range than conversational ADS. It is when CDS and conversational ADS are further compared to a third genre, MONO, which probably conflates elements of performative monologue with FDS, that sex differences in pitch use emerge. These differences could relate to: a) sex-based differences in speaking to a non-native interlocutor; b) sex-based differences in perfomative storytelling style; and/or c) possibly, heightened nervousness of the female speakers in the recorded performative context, interacting with the foreigner, which could induce higher mean pitch and greater pitch range (Fairbanks and Pronovost, 1938; Bonner, 1943; Apple et al., 1979; Laukka et al., 2008).

No significant differences for either women or men in mean F0 or F0 range between CDS to children aged 2;2–2;9 and CDS to children aged 3;0–3;10 were evident. In other words, CDS to both 2-year-olds and 3-year-olds featured higher pitch and greater pitch range than conversational ADS. Previous research (Sarvasy, 2019) suggested that a special morpho-syntactic alteration found in Nungon CDS is most prevalent in speech to children under 3;0, with phasing out thereafter; earlier studies also showed that the morpho-syntactic complexity of Nungon CDS increases from when the child is 3 years old (Sarvasy, 2019, 2020, 2021a). The current results imply that elevated mean pitch and increased pitch range may be evident in CDS even after caregivers no longer use special morpho-syntactic alterations, and have already increased the morpho-syntactic complexity of their CDS.

Much research on IDS and CDS has found that features of IDS/CDS relate to children's ages and developmental stages (Stern et al., 1983; Kitamura et al., 2002; Kitamura and Burnham, 2003; Englund and Behne, 2006; Rattanasone et al., 2013). We found no evidence that the size of the vowel triangle differs in Nungon speech directed to children in their third year of life (2;2–2;9), compared with Nungon speech directed to children in their fourth year of life (3;0–3;10). We further found no evidence that mean pitch and pitch range differ in speech to these two different age cohorts. But we did find that speech directed to the younger cohort features significantly longer vowel durations than speech directed to the older cohort, which appears similar to conversational ADS in vowel durations and duration contrasts. When only tokens from speakers featured in all three datasets are examined, both phonologically short and long vowels in CDS to 2-year-olds are significantly longer than those in MONO, which are in turn longer than those in conversational ADS and CDS to 3-year-olds. This said, we did not examine speech from a single caregiver to a single child over time to confirm these results: this longitudinal investigation remains a desideratum for future work.

Uther et al. (2007) found that vowels in British English FDS were shorter than those in IDS, as with Nungon MONO, but they found no difference in vowel length between FDS and ADS, while this does seem to exist between Nungon MONO and ADS. Again, it is as yet impossible to ascertain whether the foreigner-directed aspect of the MONO dataset induced longer vowel durations in MONO than in ADS, or whether another aspect, such as the performative context, induced this.

Burnham et al. (2002), Uther et al. (2007), Lam and Kitamura (2012), and Xu et al. (2013), among others, demonstrated, for two varieties of English, that some acoustic and prosodic modifications of speech may be predictable according to the interlocutor's relationship to the speaker and perceived linguistic abilities. For Australian and British English, the interlocutor's increased ability to provide linguistic feedback (accompanied by an apparent need for didactic intervention by the speaker) led to increased degrees of vowel hyper-articulation, while increased affect in the relationship led to increased mean pitch. The Nungon findings here from CDS and ADS do not follow the same pattern as these English studies, since Nungon CDS shows no vowel hyper-articulation, but does show increased mean pitch and pitch range. That said, the Nungon children studied here were older than the infants in the English studies. The MONO dataset shows marked vowel hyper-articulation, relative to both ADS and CDS, but it is unclear from the present study whether this stems from the identity of the interlocutor (non-native Nungon speaker with a foreign appearance) or from the particular performative context for speech.

FDS has been studied acoustically in a relatively small number of languages. The present study of acoustic properties of vowels in Nungon monologues addressed to a foreigner, compared with those of conversational, native-speaker-directed, adult speech, represents the first of which we are aware for any language of the Pacific, and for any speech community of fewer than 1 million people anywhere in the world. Like members of other small, remote speech communities (Wray and Grace, 2007), Nungon speakers do not interact with strangers on a daily basis; the very few non-native speakers of Nungon they encounter are women from nearby communities who married Nungon-speaking men. Each of the six Nungon-speaking villages has its own dialect, and Nungon further belongs to a longer dialect continuum that encompasses six additional villages, whose languages are grouped under the umbrella term “Yau” (Sarvasy, 2017). The MONO data here were addressed to one of the very few truly foreign learners of Nungon or Yau who are not native speakers of other Papuan languages spoken in Morobe Province, Papua New Guinea. One might therefore question how systematic an FDS style could be among Nungon speakers. While they do not regularly interact with foreign speakers of Nungon, Nungon speakers do regularly speak to people whose Nungon and Yau varieties differ from their own. It is unclear whether their strategies in so doing should be termed FDS. For instance, the first author observed that some speakers of the Towet village dialect of Nungon would actually assume a Kotet village-like accent and use some Kotet lexicon in interacting with speakers of the Kotet village dialect (Sarvasy, 2017).

As noted earlier, the MONO data may be hyper-articulated for a conglomeration of reasons: non-native interlocutor, high-importance recording context, and performative, rather than conversational, speech. It could be that the MONO data should be considered to represent “clear speech,” primarily (Picheny et al., 1986; Moon and Lindblom, 1994; Ferguson and Kewley-Port, 2002, 2007), and FDS only secondarily. Future work will aim to disentangle these factors. If we take the MONO results to represent FDS, however, they seem to support the notion that FDS often involves vowel hyper-articulation, and add to the complicated general picture of mean pitch and pitch range in FDS, since Nungon-speaking women and men pattern differently in how they use pitch in MONO, compared with ADS and CDS.

Acoustic research on under-described languages spoken by remote communities entails different research conditions than research with speech communities who live in proximity to university campuses with laboratory facilities. In remote areas, speech must be recorded in an outdoor or sound-permeable indoor environment, not a laboratory. Field trips to visit such communities are of limited duration and frequency. If a field trip is not devoted to a particular research question, evidence must often be culled after the trip, from an existing, multi-purpose natural speech corpus. Thus, for a study such as the present one, vowel tokens may have to be culled from uncontrolled, natural interactions in an existing speech corpus, and it may be hard to ensure that the same speakers produce vowel tokens in all three interactive contexts under study. In studying child language development in such a community, researchers may end up devoting all resources into building a corpus of child-caregiver speech, without also constructing a counterpart corpus of the caregivers addressing other adults in a similar, informal and conversational, vein. A child-caregiver corpus will yield ample tokens of CDS vowels and consonants, but the researcher may not have access to optimal ADS tokens for comparison.

In recent work, some researchers have chosen to compare features of conversational CDS to a corpus of non-conversational adult speech. For instance, Frye aus Schwerte (2019) compares prosodic features of CDS in the Papuan language Qaqet to those in adult “Pear Story” narratives, rather than truly conversational adult speech. Our first exploratory comparative study of Nungon CDS and ADS compared CDS directly to the narrative monologue data in the MONO dataset here (Sarvasy et al., 2019). We resorted to this because we had not yet commissioned the dyadic ADS conversations, with no foreigners present, described in the present study.

The results here indicate that, difficult as it may be to source conversational ADS to compare to CDS in languages spoken by small, remote communities, this is just as important for these languages as it is for languages like Japanese (Miyazawa et al., 2017). The marked contrast in vowel space area between MONO, ADS, and CDS shows that even in a community without a tradition of literacy, vowel acoustics differ in different contexts and settings: here, speech addressed to children, conversation among familiars, and a more presentative mode with a non-native interlocutor. Future research would do well to consider this carefully.

## Conclusion

This study has shown for the Towet village dialect of the Papuan language Nungon that vowels in speech directed to children aged 2;2–3;10 are not hyper-articulated, compared with speech directed to adults. Nungon speech directed to children of this age by both women and men features higher mean pitch and increased pitch range, compared with conversational speech directed to adults. Speech directed to 2-year-olds (2;2–2;9) features similar vowel space areas (vowel triangle sizes), mean pitch, and pitch range, to speech directed at 3-year-olds (3;0–3;10); this holds for both men and women. Speech directed at 2-year-olds features significantly longer vowels than speech directed at 3-year-olds, in which vowel length is similar to that of conversational adult-directed speech. The duration distinction between phonologically short and phonologically long Nungon vowels is upheld in speech to 2-year-olds, 3-year-olds, and adults. Compared with both child-directed speech and conversational adult-directed speech, monologues directed at a non-native Nungon speaker feature marked vowel hyper-articulation. The relationship between mean pitch and pitch range in these foreigner-directed monologues and those in child-directed and adult-directed conversational speech differed for men and women. This has been the first study comparing vowel acoustics and pitch in child-directed speech and adult-directed speech for a language of the New Guinea area, where 20% of the world's languages are spoken; it is further the first study comparing vowels in child-directed speech, adult-directed speech, and another clear speech style for a small language community.

## Data availability statement

The datasets analyzed for this study can be found in the Open Science Framework site at: https://osf.io/6pr8s/.

## Ethics statement

The studies involving human participants were reviewed and approved by the Australian National University and Western Sydney University Human Research Ethics Committees. Written informed consent to participate in this study was provided by the participants' legal guardian/next of kin.

## Author contributions

HSS collected data, drafted, and revised the manuscript. WL ran analyses, helped draft, and revised the manuscript. JE assisted with revising the manuscript. PE assisted with revising the manuscript. All authors contributed to the article and approved the submitted version.

## Funding

Funding was received from: the Australian Research Council (Grants CE140100041 and DE180101609 to HSS and FT160100514 to PE), the MARCS Institute for Brain, Behavior and Development, and the ARC Centre of Excellence for the Dynamics of Language (Language Documentation grant to HSS).

## Conflict of interest

The authors declare that the research was conducted in the absence of any commercial or financial relationships that could be construed as a potential conflict of interest.

## Publisher's note

All claims expressed in this article are solely those of the authors and do not necessarily represent those of their affiliated organizations, or those of the publisher, the editors and the reviewers. Any product that may be evaluated in this article, or claim that may be made by its manufacturer, is not guaranteed or endorsed by the publisher.
